# How Native and Alien Metal Cations Bind ATP: Implications for Lithium as a Therapeutic Agent

**DOI:** 10.1038/srep42377

**Published:** 2017-02-14

**Authors:** Todor Dudev, Cédric Grauffel, Carmay Lim

**Affiliations:** 1 Faculty of Chemistry and Pharmacy, Sofia University, Sofia 1164, Bulgaria; 2 Institute of Biomedical Sciences, Academia Sinica, Taipei 11529, Taiwan; 3 Department of Chemistry, National Tsing Hua University, Hsinchu 300, Taiwan

## Abstract

Adenosine triphosphate (ATP), the major energy currency of the cell, exists in solution mostly as ATP-Mg. Recent experiments suggest that Mg^2+^ interacts with the highly charged ATP triphosphate group and Li^+^ can co-bind with the native Mg^2+^ to form ATP-Mg-Li and modulate the neuronal purine receptor response. However, it is unclear how the negatively charged ATP triphosphate group binds Mg^2+^ and Li^+^ (i.e. which phosphate group(s) bind Mg^2+^/Li^+^) and how the ATP solution conformation depends on the type of metal cation and the metal-binding mode. Here, we reveal the preferred ATP-binding mode of Mg^2+^/Li^+^ alone and combined: Mg^2+^ prefers to bind ATP tridentately to each of the three phosphate groups, but Li^+^ prefers to bind bidentately to the terminal two phosphates. We show that the solution ATP conformation depends on the cation and its binding site/mode, but it does not change significantly when Li^+^ binds to Mg^2+^-loaded ATP. Hence, ATP-Mg-Li, like Mg^2+^-ATP, can fit in the ATP-binding site of the host enzyme/receptor, activating specific signaling pathways.

Lithium (Li^+^), a non-biogenic cation not known to have essential biological functions in mammals, is used (in the form of soluble salts) as a first-line medication for psychiatric diseases, in particular bipolar disorder^1^. It has been considered as a possible treatment for chronic neurodegenerative diseases such as Alzheimer’s, Parkinson’s, and Huntington’s^1^,^2^. Although the beneficial effects of Li^+^ therapy have been known for decades, the mechanism of Li^+^ action remains largely enigmatic. Several hypotheses have been put forth which, taken together, suggest that Li^+^ may exert its therapeutic effect via diverse, multifaceted pathways:

1. *Li*^+^ *competes with Na*^+^ *in the cytosol.* Li^+^ enters the intracellular space via sodium channels or transporters and accumulates, thus competing with Na^+^. In the cytosol, elevated Li^+^ concentration decreases the Na^+^ level, which in turn reduces the Ca^2+^ concentration^3^. Lowering both cytosolic Na^+^ and Ca^2+^ concentrations decreases the cell excitability and eventually normalizes the neuron activity in bipolar disorder patients whose intracellular Na^+^ concentration is abnormally high^3^,^4^.
2. *Li*^+^ *modulates neurotransmitter signaling*. Lithium may interact with cellular receptors that regulate the synthesis, release, turnover and reuptake of neurotransmitters such as dopamine and serotonin. Thus, lithium’s therapeutic effect has been postulated to be related to its ability to modulate neurotransmitter signaling in the central nervous system^5^.
3. *Li*^+^ *competes with Mg*^*2*+^ *for specific protein binding sites.* This hypothesis posits that Li^+^, by replacing the native Mg^2+^ cofactor, inhibits key metalloenzymes (G-proteins, GSK-3β, inositol monophosphatase, inositol polyphosphate phosphatase) involved in specific neurotransmission pathways in the brain^6^,^7^,^8^.
4. *Li*^+^ *affects signaling pathways involving Mg*^*2*+^*-loaded nucleotide cofactors*. Li^+^ has been hypothesized to co-bind with Mg-bound adenosine triphosphate (ATP) forming a ATP-Mg-Li complex which, when protein-bound, may elicit different responses from key ATP-dependent enzymes/receptors involved in cell signaling^9^.

The last hypothesis is supported by recent experiments showing that the ATP-Mg-Li complex can indeed modulate the neuronal purine receptor response^10^. The P2X receptor, a ligand-gated ion channel that mediates the influx of extracellular Ca^2+^ into the cytoplasm, exhibited prolonged activation when stimulated by ATP-Mg-Li. Solution ^7^Li and ^31^P NMR experiments show that upon the metal–ATP complexation, Mg^2+^ and Li^+^ interact with the highly charged triphosphate group and do not bind the adenine and ribose moieties. Notably, Li^+^ does not compete with Mg^2+^ for the ATP-binding site(s); instead it binds to the Mg^2+^-loaded ATP forming a ternary ATP-Mg-Li complex. The interactions between the metal cation and ATP are thought to be mostly electrostatic, guided by the cation’s coordination preference.

Although ATP is known to exist as [ATP-Mg]^2−^ in neutral solution^11^,^12^, a solution structure of Mg^2+^-bound ATP has remained elusive. There is also no solution structure of Li^+^ bound to free or Mg^2+^-bound ATP. Thus, the most stable binding mode of Mg^2+^/Li^+^ to ATP or Li^+^ to [ATP-Mg]^2−^ in solution remains unclear, raising several intriguing and quite fundamental questions:

1. Which of the three ATP phosphate sites (labeled α, β, and γ in order of increasing distance from the ribose) does Mg^2+^ prefer and does it prefer to bind to one (monodentate) phosphate O or simultaneously to two (bidentate) or three (tridentate) phosphate O atoms?
2. Does Li^+^ show the same ATP-binding mode preference as Mg^2+^?
3. When Mg^2+^ is already bound to ATP, which phosphate(s) best accommodate Li^+^ binding?
4. How is the ATP solution conformation affected by metal ion binding? Is the native ATP-Mg conformation altered by Li^+^ binding, thus affecting enzyme/receptor recognition?

To address these questions, we modeled Li^+^/Mg^2+^-ATP complexes with different metal composition, coordination sites, and metal-binding modes (mono/bi/tridentate), and evaluated their thermodynamic characteristics using density functional theory combined with a polarizable continuum model (see Methods). First, the calculations were calibrated with respect to available experimental data. They reveal the relative stabilities of Li^+^/Mg^2+^-ATP complexes that differ in metal-binding mode and how the ATP conformation differs depending on the cation type (Mg^2+^/Li^+^) and the metal-binding mode. Importantly, the results show that when Li^+^ co-binds with Mg^2+^ to the ATP triphosphate group, the native ATP-Mg conformation remains virtually unchanged. Thus, like native ATP-Mg, the ATP-Mg-Li complex may also be bound by cellular receptors or ATP-dependent enzymes and activate specific signaling pathways.

## Results

### Effect of Mg^2+^-Binding Mode on the ATP Conformation

**Figure 1:**
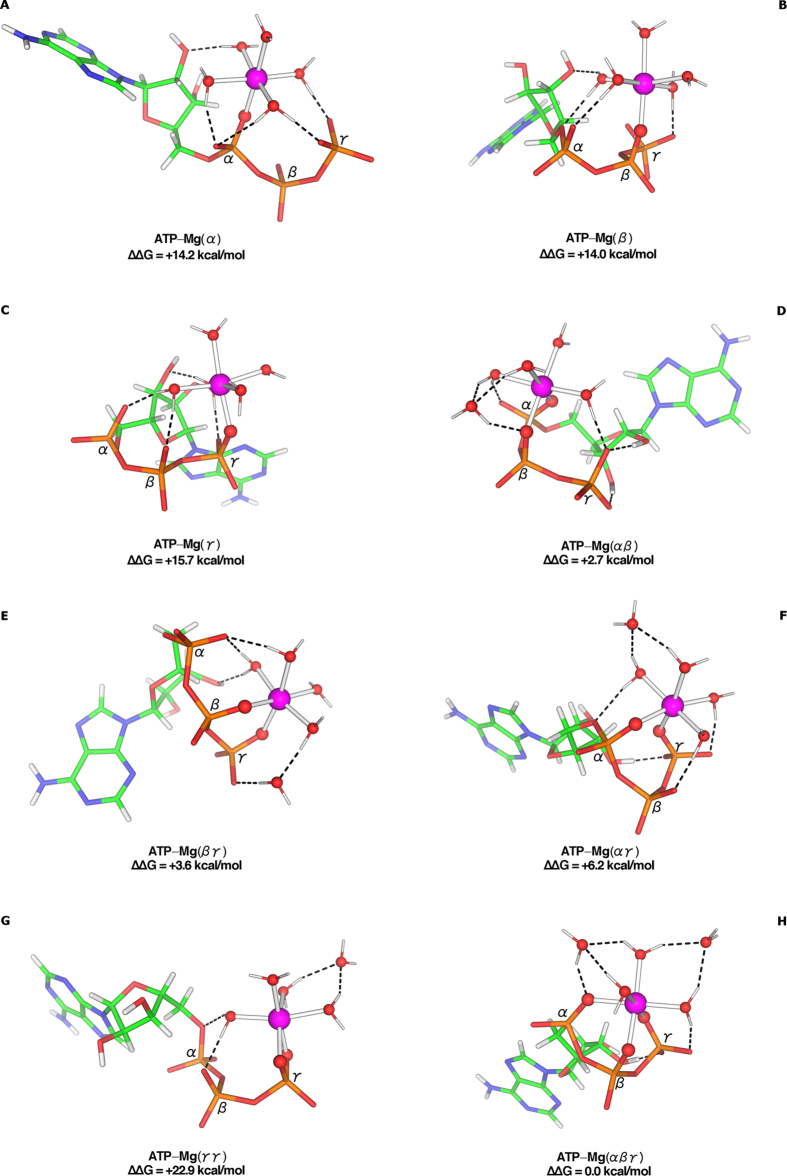
M062X/6-311++G(d,p) optimized structures of [ATP-Mg]^2−^ complexes and relative free energies, ∆∆*G* (kcal/mol), of complex formation in water. The dashed lines indicate hydrogen bonds, defined by a hydrogen-acceptor distance <1.4 Å and a D–H…A angle >130°. The structures were oriented to give the clearest view of the metal-binding site.

Each of the ATP phosphate groups (α, β, and γ) was probed for its ability to bind Mg^2+^ by itself or in combination with its neighbor(s). The fully optimized structures of Mg^2+^ bound (i) *mono*dentately to the α/β/γ phosphate (Fig. 1A–C), (ii) *bi*dentately to the αβ, βγ, αγ, and γγ phosphates (Fig. 1D–G), and (iii) *tri*dentately to αβγ phosphates (Fig. 1H) are stabilized by favorable metal–O(phosphate) charge–charge interactions and water···O(ribose/phosphate) hydrogen bonds. They reveal that ATP, when allowed to freely optimize its geometry in water, adopts distinct conformations depending on the metal-binding mode/site. The mean root-mean-square deviation (RMSD) between the ATP heavy atoms of any two superimposed structures in Fig. 1 is 1.9 Å with the largest RMSD (3.4 Å) between the βγ and α structures, and the next largest RMSD (3.1 Å) between the βγ and γγ structures (Supplementary Table S1a). This underscores the importance of the metal-binding mode and site on the nucleotide conformation.

### Mg^2+^ Prefers to Bind Tridentately to all 3 ATP Phosphates

The free energy of ATP-Mg complex formation relative to the free energy of the αβγ tridentate complex show that Mg^2+^ prefers multidentate to monodentate binding: Compared to the αβγ tridentate complex, the complexation free energies are less favorable by 3, 4, and 6 kcal/mol for the αβ, βγ, and αγ bidentate structures, respectively and by 14–16 kcal/mol for the monodentate complexes (see Fig. 1). The least preferred metal-binding mode corresponds to Mg^2+^ bound to two O atoms from the same (γ) phosphate (Fig. 1g). This is likely due to the unfavorable coordination geometry imposed on Mg^2+^ in this binding mode: the O^P^–Mg–O^P^ angle (72°) is more acute than the mean O^P^–Mg–O^P^ angle (~91°) in the other polydentate structures, which corresponds to the preferred coordination geometry of Mg^2+ ^13. For the same reason, a ATP–Mg(βγγ) tridentate structure (see Supplementary Figure S1), which had been used to study ATP hydrolysis^14^, was disfavored (by 11 kcal/mol) compared to the ATP–Mg(αβγ) tridentate structure (Fig. 1H). Thus, Mg^2+^ favors binding all three ATP phosphate groups forming two six-membered rings, thus stabilizing the ATP–Mg(αβγ) tridentate structure.

### Effect of Li^+^-Binding Mode on the ATP Conformation

**Figure 2:**
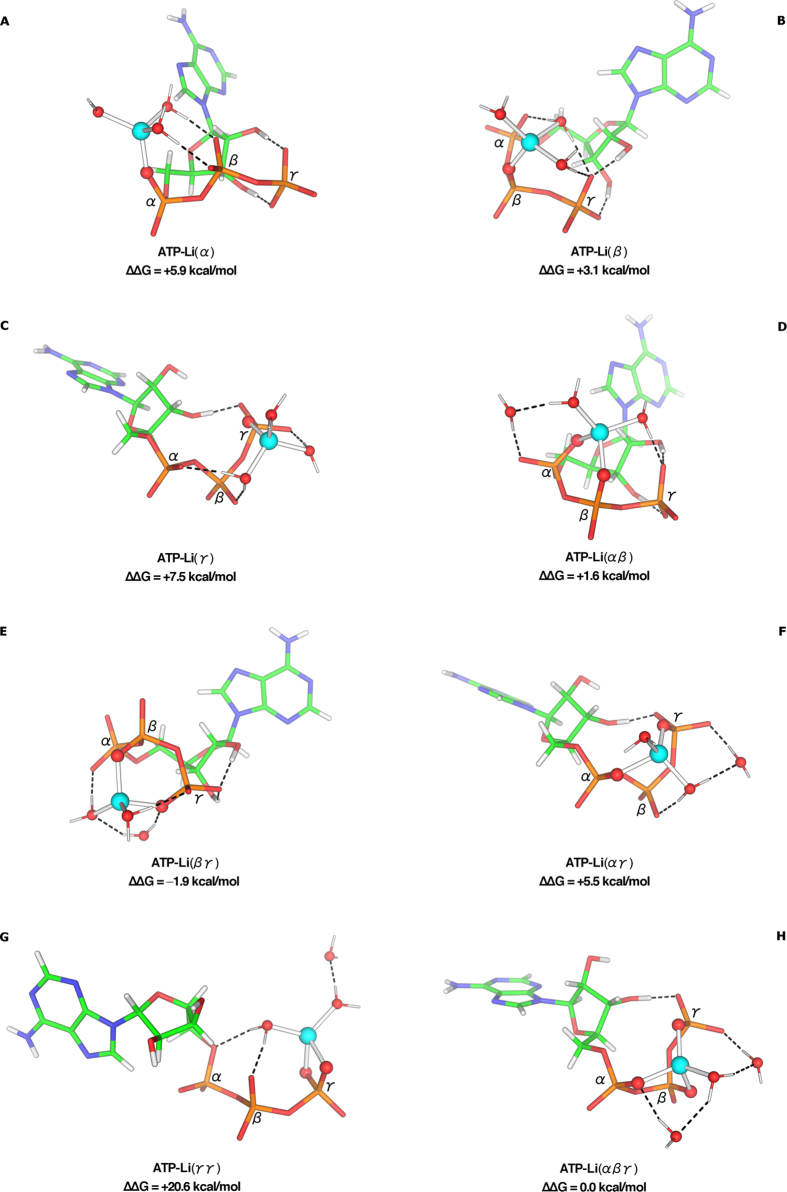
M062X/6-311++G(d,p) optimized structures of [ATP-Li]^3−^ complexes and relative free energies, ∆∆*G* (kcal/mol), of complex formation in water.

To examine if ATP binds Li^+^ in the same way as Mg^2+^ and how its conformation depends on the Li^+^-binding mode, we fully optimized Li^+^ counterparts of the Mg-ATP complexes (Fig. 2A–H). Compared to Mg^2+^, the weaker coordination strength of Li^+^ induces smaller conformational changes: only two pairs of ATP-Li structures exhibit RMSD ≥2 Å, compared with ten pairs of ATP-Mg structures (Supplementary Table S1b). Depending on the ATP-binding mode, Li^+^ and Mg^2+^ induce different changes in the nucleotide conformation: The RMSDs between the ATP heavy atoms of superimposed [ATP-Mg]^2−^ and [ATP-Li]^3−^ α, γ, β, and βγ structures are respectively 1.7, 1.8, 2.0, and 2.6 Å, but are ≤0.6 Å for the other bi/tridentate-binding modes.

### Li^+^ Prefers to Bind Bidentately to the ATP βγ Phosphates

Whereas the tridentate ATP-Mg structure (Fig. 1H) is the most stable, upon Li^+^ binding to ATP, the bidentate βγ complex (Fig. 2E) is slightly more stable than the tridentate complex (Fig. 2H), which is more stable than the other bidentate or monodentate structures. Like Mg^2+^, Li^+^ bound to two O atoms from the same phosphate group (Fig. 2G) is energetically unfavorable, as this binding mode creates coordination geometry strain with a O^P^–Li–O^P^ angle (78°) much smaller than the mean O^P^–Li–O^P^ angle (~105°) in the other bi/tridentate structures. Whereas divalent Mg^2+^ exhibits distinct preference towards the ATP-binding sites, monovalent Li^+^ appears less discriminative: Excluding the high-energy γγ configuration (Figs 1G and 2G), the ΔΔG range for the ATP-Mg complexes (~16 kcal/mol) is greater that for the ATP-Li structures (~10 kcal/mol).

### Li^+^ Binds Mg-bound ATP Forming a OH-Bridged Binuclear Complex

**Figure 3:**
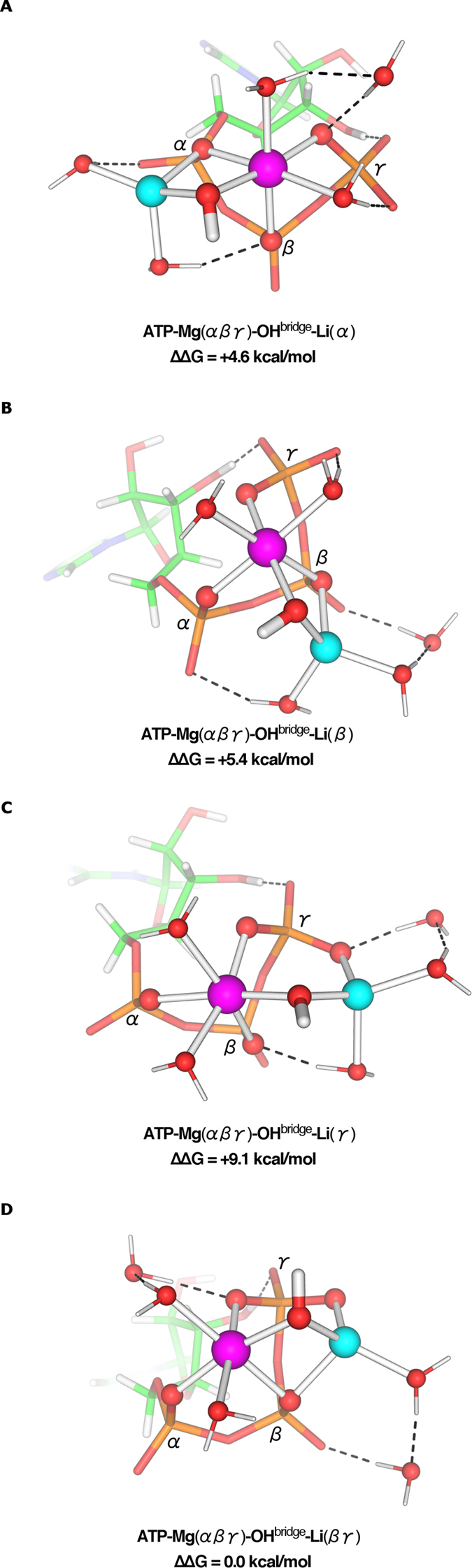
M062X/6-311++G(d,p) optimized structures of Li^+^ bound to ATP-Mg(αβγ), and relative free energies, ∆∆G (kcal/mol), of complex formation in water.

The most stable and hence most populated [ATP-Mg]^2−^ complex in solution with Mg^2+^ bound to ATP tridentately (Fig. 1H) was used to derive bimetallic [ATP-Mg-Li]^−^ complexes where different available sites were systematically probed for their varying Li^+^ affinities (Fig. 3). Due to the structural constraints imposed by Mg^2+^ binding to all three ATP phosphates and because the γγ mode (Fig. 2G) is a high-energy configuration, we modeled Li^+^ binding mono/bidentately to the ATP triphosphate group, yielding a mono or binuclear site with a water molecule bridging the two cations. As shown in our previous work^15^, the bridging water molecule may be deprotonated; hence, its protonation state was determined by computing the ∆*G*^*deprot*^ free energy for **ATP**-**Mg(αβγ)**-**H**_**2**_**O**^**bridge**^-**Li** + OH^−^ → **ATP**-**Mg(αβγ)**-**OH**^**bridge**^-**Li **+** **H_2_O in solution. Regardless of the coordination mode, the resulting ∆*G*^*deprot*^ is negative (−16 to −20 kcal/mol). Even though the hydroxide concentration is minute (10^−7^ M) at physiological pH of 7, a hydroxide bridge is still favored over a water bridge; e.g., the ∆*G*^*deprot*^ for **ATP**-**Mg(αβγ)**-**H**_**2**_**O**^**bridge**^-**Li(βγ) **+** **OH^−^ → **ATP**-**Mg(αβγ)**-**OH**^**bridge**^-**Li(βγ) **+** **H_2_O of −16 kcal/mol yields a concentration of the **OH**^**bridge**^ complex that is ~1,000 greater than that of the **H**_**2**_**O**^**bridge**^ counterpart. Furthermore, the **OH**^**bridge**^ structures in Fig. 3 were more stable than their non-bridged counterparts (Supplementary Figure S2).

### Li^+^ Prefers to Bind Bidentately to Mg-Bound ATP

Among the complexes formed by Li^+^ binding to the tridentate **ATP**-**Mg(αβγ)** structure, the most stable one corresponds to Li^+^ bidentately bound to the β and γ phosphates (Fig. 3D), as found for Li^+^ binding to free ATP. In this **ATP**-**Mg(αβγ)**-**OH**^**bridge**^-**Li(βγ)** structure, the two metal ions and the bridging hydroxide form a four-membered ring with the β phosphate and a six-membered ring with the **γ** phosphate. Such a bicyclic structure cannot be formed when Li^+^ is monodentately bound to the ATP: Li^+^ forms a four or six-membered ring in the monodentate structures (Fig. 3A–C), which are less stable than the **ATP**-**Mg(αβγ)**-**OH**^**bridge**^-**Li(βγ)** bidentate structure (by ~5–9 kcal/mol).

**Figure 4:**
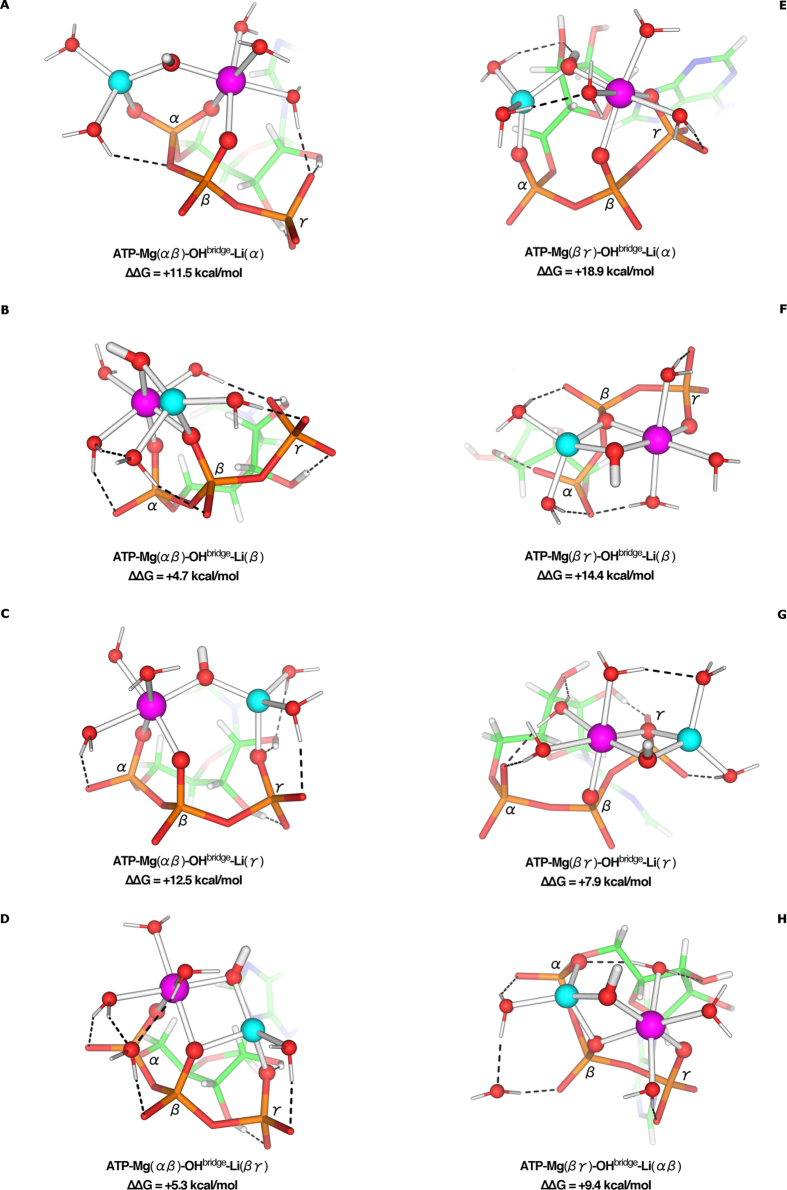
M062X/6-311++G(d,p) optimized structures of Li^+^ bound to ATP-Mg(αβ) (left) or ATP-Mg(βγ) (right) and relative free energies, ∆∆G (kcal/mol), of complex formation in water.

Since **ATP-Mg**-**αβ** and **ATP-Mg**-**βγ** (Fig. 1D,E) have comparable stabilities and are the next most stable (populated) [ATP-Mg]^2−^ conformers in solution, they were also used to derive bimetallic [ATP-Mg-Li]^−^ complexes. The fully optimized solution structures of the [ATP-Mg-Li]^−^ complexes with a bridging hydroxide (Fig. 4) are far more stable than the corresponding structures without a bridging hydroxide (Supplementary Figure S3). Relative to the free energy of the **ATP**-**Mg(αβγ)**-**OH**^**bridge**^-**Li(βγ**) structure (Fig. 3D), the complexes formed by Li^+^ binding to the bidentate **ATP**-**Mg(αβ)** or **ATP**-**Mg(βγ)** structure are all less stable (by 5–19 kcal/mol, Fig. 4) probably because they cannot form a bicyclic structure as for the **ATP**-**Mg(αβγ)**-**OH**^**bridge**^-**Li(βγ**) complex.

### Li^+^ Does Not Significantly Alter the Mg-Bound ATP Conformation

As the triphosphate moiety conformation has been firmly locked by bi/tridentate coordination of Mg^2+^, the ATP overall conformation remains virtually unchanged upon Li^+^ binding, regardless of its coordination mode/site. The RMSD of the ATP heavy atoms in the [ATP-Mg-Li]^−^ structures (Figs 3 and 4) from those in the [ATP-Mg]^2−^ counterparts are generally ≤0.6 Å (see Supplementary Table S2), which is within the RMSD resulting from thermal fluctuations.

## Discussion

**Figure 5:**
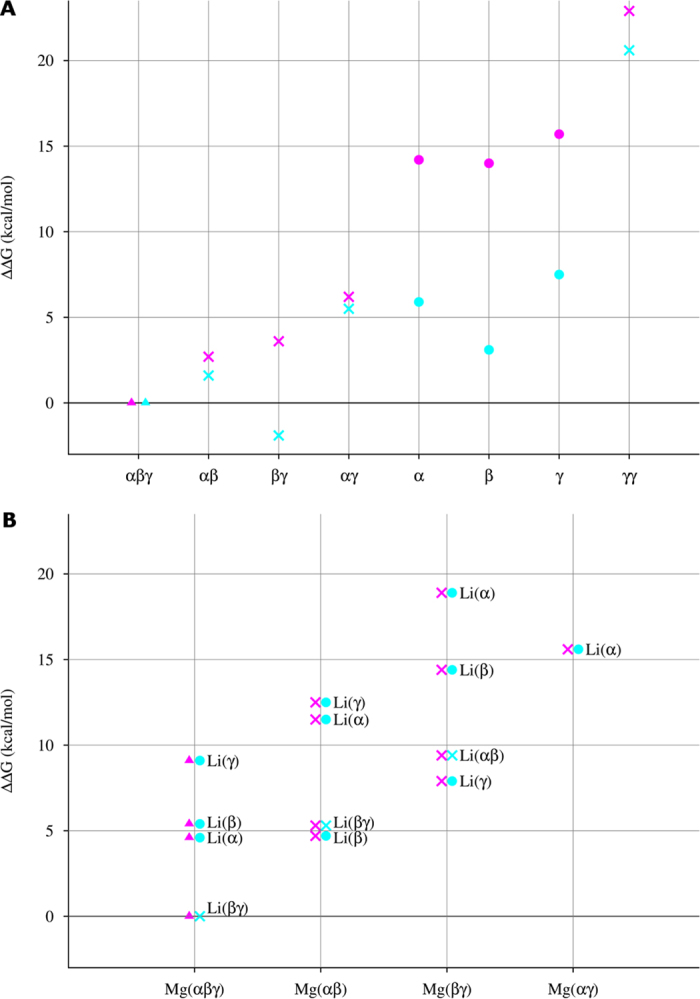
Relative solution free energies, ∆∆*G* (kcal/mol), of (**A**) [ATP-Mg]^2−^ (magenta) and [ATP-Li]^3−^ (turquoise) complexes from Figs 1 and 2, respectively, and (**B**) [ATP-Mg-Li]^−^ complexes from Figs 3 and 4 with the Mg- and Li-binding modes in magenta and turquoise, respectively. The filled circles, crosses and triangles denote mono, bi and tridentate binding, respectively.

Despite ATP’s importance as the major energy currency of the cell and its known existence in solution mostly as [ATP-Mg]^2−^, how its triphosphate group binds the native Mg^2+^ ion or alien cations such as Li^+^ and how its solution conformation depends on the metal ion type and metal-binding mode was not known. By computing the solution structures and free energies of various [ATP-Mg]^2−^, [ATP-Li]^3−^, and [ATP-Mg-Li]^−^ complexes differing in metal-binding mode, as summarized in Fig. 5, we have delineated the most thermodynamically preferred structures, which result mainly from a balance between “intramolecular” phosphate–metal/water interactions and “intermolecular” phosphate–solvent interactions. We show that in solution, ATP prefers to bind Mg^2+^ via all three αβγ phosphates, but it prefers to bind Li^+^ via its terminal βγ phosphates. We also show that in solution, Mg-bound ATP binds Li^+^ bidentately to form a OH-bridged **ATP**-**Mg(αβγ)**-**OH**^**bridge**^-**Li(βγ)** complex (Fig. 3D).

The lowest free-energy solution structures of ATP-Mg (Fig. 1H) and ATP-Li (Fig. 2E) complexes yielded a free energy for [Mg(H_2_O)_6_]^2+^ + [Li (H_2_O)_3_ ATP]^3−^ → [Mg (H_2_O)_5_ ATP]^2−^ + [Li(H_2_O)_4_]^+^ (−4.2 kcal/mol, see Methods) in agreement with the corresponding experimental value (−3.5 kcal/mol^12^), thus lending support to the preferred binding modes found herein. Our finding that tridentate coordination of Mg^2+^ to ATP is slightly favored over αβ bidentate coordination is consistent with experimental estimates of ~60% tridentate coordination for [ATP-Mg]^2−^ in solution^16^. That Li^+^ and Mg^2+^ prefers to ligate to two and three phosphate O atoms, respectively, is in line with earlier work showing that the maximum number of metal-bound anionic O-containing ligands is two for a monocation, but three for a dication^17^. Notably, in a previous study^10^, a **ATP**-**Mg(βγ)**-H_2_O^**bridge**^-**Li(γ**) structure was proposed for the [ATP-Mg-Li]^−^ complex. This structure was found herein to be high-energy one: It is less stable than the corresponding hydroxide-bridged structure (Fig. 4G), which in turn is less stable than the **ATP**-**Mg(αβγ)**-**OH**^**bridge**^-**Li(βγ**) structure by ~8 kcal/mol.

We also reveal how the metal cation type and its binding mode affect the ATP conformation. Li^+^ binding to Mg^2+^-loaded ATP did not significantly alter the ATP conformation or the properties of the P^**γ**^–O(–P^**β**^) bond that is hydrolyzed: The P–O bond lengths in the Mg-ATP and Mg-Li-ATP complexes are identical (1.693 Å), while the bond polarities, estimated by the difference between the P and O Hirschfeld charges, are 0.81e and 0.83e, respectively. These findings have important consequences for [ATP-Mg]^2−^ and [ATP-Mg-Li]^−^ recognition by cellular receptors/ATP-dependent enzymes. Since these two types of metal complexes have similar overall ATP conformation and P^**γ**^–O(–P^**β**^) bond properties, the [ATP-Mg-Li]^−^ complex might fit in the host receptor/enzyme binding site and trigger cellular response. Indeed, experiments show that [ATP-Mg-Li]^−^, like the native [ATP-Mg]^2−^ complex, is recognized by purinergic receptors and can activate subsequent signaling pathways^10^. Hence, Li^+^ binding to Mg^2+^-loaded ATP may permit recognition of the [ATP-Mg-Li]^−^ complex by certain host enzymes/receptors and activate specific signaling pathways. These findings thus help elucidate the mechanism of lithium’s therapeutic action.

## Methods

### Modeling ATP Complexes

As the pK_a_ of ATP ranges from 6.5–6.95^18^,^19^, its dominant form at physiological pH is ATP^4−^. Furthermore, ATP exists mostly as [ATP-Mg]^2−^ in neutral solution^11^. Since Mg^2+^ is mostly hexacoordinated in complexes with organic ligands and proteins^20^,^21^, as in aqueous solution^22^, hexacoordinated [Mg(H_2_O)_5_.ATP]^2−^ complexes were modeled. In contrast to Mg^2+^, Li^+^ is found mostly tetracoordinated^21^,^23^, hence its complexes with ATP were modeled as [Li(H_2_O)_3_.ATP]^3−^ or [Mg(H_2_O)_5_.ATP.Li(H_2_O)_3_]^−^. Regardless of the metal-binding mode, the number of water molecules (five for Mg^2+^ and three for Li^+^ complexes) was kept the same: all water molecules were bound directly to the cation in monodentate complexes, but one and two water molecules were transferred to the second shell in bidentate and tridentate complexes, respectively. All the structures were built using GaussView version 3.09^24^.

### Geometry Optimization

High-resolution structures of pertinent Mg^2+^ and Li^+^ complexes from the Cambridge Structural Database^25^ (see Table 1) were used to determine an optimal method for optimizing the geometries of Mg^2+^ and Li^+^ complexes. Among the various combinations of different density functionals (B3LYP, SVWN, M062X, M06HF and BMK) and basis sets (6-31 + G(d), 6-31 + G(d,p), 6-31 + G(2d,2p), 6-31 + G(3d,p), 6-31 + G(3d,2p), 6-311++G(d,p), 6-311++G(2d,2p)) tested, the M062X/6-311++G(d,p) method was found to be the most efficient in yielding structural parameters of Mg^2+^ and Li^+^ complexes that are closest to the respective experimental values (see Table 1 and Supplementary Tables S3a–e).

Hence, the M062X/6-311++G(d,p) method was used to optimize the geometry of each ATP-Mg^2+^/Li^+^ complex in water employing the polarizable continuum model implemented in Gaussian 09^26^ and to compute the respective vibrational frequencies. For each metal-binding mode/site, we modeled many structures, trying to maximize the number of water–phosphate and water–ribose/water hydrogen-bonding interactions. The optimized complex with the lowest energy was chosen for further evaluations (see below) – no imaginary frequency was found in the chosen complexes.

### Solution Free Energy Calculation

The electronic energies in solution, *E*_el_, were corrected by single-point energy calculations implementing the SMD solvation model^27^. The thermal energies (*E*_th_) and entropies (*S*) were computed from standard statistical mechanical formulas^28^ using frequencies scaled by an empirical factor of 0.979^29^. The differences ∆∆*E*_el_, ∆∆*E*_th_, and ∆∆*S* between the respective metal complexes were used to calculate the relative formation free energies, Δ∆*G*, at *T* = 298.15 K according to


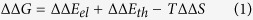


The experimental binding constants of ATP-Mg (9554 M^−1^) and ATP-Li (25 M^−1^) complexes^12^ were used to determine an optimal method for the single-point energy calculations. Since it is unclear if one or two Li^+^ ions are bound to ATP, both [Li(H_2_O)_3_ATP]^3−^ and [Li_2_(H_2_O)_6_ATP]^2−^ were modelled. The lowest free-energy structures of ATP-Mg (Fig. 1), ATP-Li (Fig. 2), and Li_2_ATP (Supplementary Figure S4) complexes were used to compute the solution free energy for replacing Li^+^ bound to ATP with Mg^2+^. Using single-point M062X/6-311++G(d,p) calculations, the solution free energy for [Li_2_(H_2_O)_6_ATP]^2−^ + [Mg(H_2_O)_6_]^2+^ + H_2_O → [Mg(H_2_O)_5_ATP]^2−^+2[Li(H_2_O)_4_]^+^ was computed to be 18.5 kcal/mol for a hydroxide molecule bridging the two Li^+^ ions. It remained positive (7.3 kcal/mol) even if a water molecule replaced the bridging hydroxide. On the other hand, the solution free energy for [Li(H_2_O)_3_ATP]^3−^ + [Mg(H_2_O)_6_]^2+^ → [Mg(H_2_O)_5_ATP]^2−^ + [Li(H_2_O)_4_]^+^ (−5.5 kcal/mol) is close to the respective experimental free energy (−3.5 kcal/mol), indicating that only one Li^+^ is likely bound to ATP.

As the M062X/6-311++G(d,p) energies overestimated the experimental free energy, single-point calculations were performed using M062X with increasing basis sets (6-311++G(2d,2p) 6-31 + G(3d,p), 6-311++G(3d,p) as well as B3-LYP with D3 dispersion correction (B3LYP-D3). With increasing basis set, the solution free energy for [Li(H_2_O)_3_ATP]^3−^ + [Mg(H_2_O)_6_]^2+^ → [Mg(H_2_O)_5_ATP]^2−^ + [Li(H_2_O)_4_]^+^ converged to a value roughly twice the experimental number using M062X, but to within 1.5 kcal/mol of the experimental free energy using B3LYP-D3 (Table 2). Since the B3LYP-D3/6-311++G(d,p) method could reproduce the experimental Li^+^ → Mg^2+^ exchange free energy in solution to within a kcal/mol, it was chosen for all single-point energy calculations.

## Additional Information

**How to cite this article**: Dudev, T. *et al*. How Native and Alien Metal Cations Bind ATP: Implications for Lithium as a Therapeutic Agent. *Sci. Rep.* **7**, 42377; doi: 10.1038/srep42377 (2017).

**Publisher's note:** Springer Nature remains neutral with regard to jurisdictional claims in published maps and institutional affiliations.

## Supplementary Material

Supplementary Information

## Figures and Tables

**Table 1:**
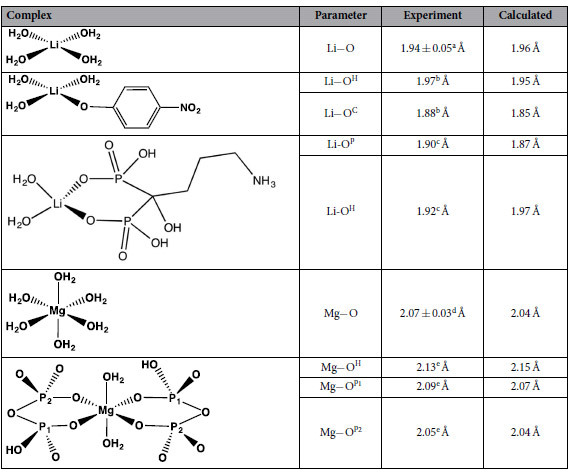
Comparison Between Computed and Experimental Average Metal−O Distances of M062X/6-311++G(d,p)-Optimized Mg^2+^ and Li^+^ Complexes.

^a^From Dudev & Lim, 2006^17^. ^b^Cambridge Structural Database entry, FECWIT. ^c^Cambridge Structural Database entry, EJEZUP. ^d^From Dudev & Lim, 2005^30^. ^f^Cambridge Structural Database entry, GEQBIO.

**Table 2:** Computed Solution Free Energies for [Li(H_2_O)_3_ATP]^3−^+[Mg(H_2_O)_6_]^2+^ → [Mg(H_2_O)_5_ATP]^2−^+[Li(H_2_O)_4_]^+^ using Different Methods^a^.

Basis Set	6-311 + G(d)	6-311++G(d)	6-311++G(d,p)	6-311++ G(2d,2p)	6-31 + G(3d,p)	6-311++G(3d,p)
# of functions	848	866	920	1149	1187	1270
M062X	−3.7	−3.6	−5.5	−6.4	−6.7	−6.9
B3LYP-D3	−2.6	−2.4	−**4.2**	−4.6	−5.0	−5.1

^a^Experimental value of −3.5 kcal/mol is determined from the binding constants of ATP-Mg (9554 M^−1^) and ATP-Li (25 M^−1^) complexes from Wilson and Chin, 1991^12^.

## References

[b1] Marmol F. Lithium: Bipolar disorder and neurodegenerative diseases. Possible cellular mechanisms of the therapeutic effects of lithium. Progr. Neuro-Psychopharmacol. Biol. Psychiatry 32, 1761–1771 (2008). 10.1016/j.pnpbp.2008.08.012 18789369

[b2] Chiu C.-T. & Chuang D.-M. Molecular actions and therapeutic potential of lithium in preclinical and clinical studies of CNS disorders. Pharmacology & Therapeutics 128, 281–304 (2010). 20705090 10.1016/j.pharmthera.2010.07.006 PMC3167234

[b3] Huang X., Lei Z. & El-Mallakh R. S. Lithium normalizes elevated intracellular sodium. Bipolar Disord. 9, 298–300 (2007). 17430305 10.1111/j.1399-5618.2007.00429.x

[b4] El-Mallakh R. S. Ion homeostasis and the mechanism of action of lithium. Clin. Neurosci. Res. 4, 227–231 (2004).

[b5] Chenu F. & Bourin M. Potentiation of antidepressant-like activity with lithium: mechanism involved. Curr. Drug Targets 7, 159–163 (2006). 16475957 10.2174/138945006775515392

[b6] Quiroz J. A., Machado-Vieira R., Zarate C. A. Jr. & Manji H. K. Novel insights into lithium’s mechanism of action: neurotrophic and neuroprotective effects. Neuropsychobiology 62, 50–60 (2010). 20453535 10.1159/000314310 PMC2889681

[b7] Dudev T. & Lim C. Competition between Li^+^ and Mg^2+^ in Metalloproteins. Implications for Lithium Therapy. J. Am. Chem. Soc. 133, 9506–9515 (2011). 21595457 10.1021/ja201985s

[b8] Brown K. M. & Tracy D. K. Lithium: the pharmacodynamic actions of the amazing ion. Ther. Adv. Psychopharmacol. 3, 163–176 (2013). 24167688 10.1177/2045125312471963 PMC3805456

[b9] Birch N. J. Possible mechanism for biological action of lithium. Nature 264, 681–681 (1976). 10.1038/264681a0 1004615

[b10] Briggs K. T., Giulian G. G., Li G., Kao J. P. Y. & Marino J. P. A molecular model for lithium’s bioactive form. Biophys. J. 111, 294–300 (2016). 27463132 10.1016/j.bpj.2016.06.015 PMC4968421

[b11] Storer A. C. & Cornish-Bowden A. Concentration of MgATP^2−^ and other ions in solution. Biochem. J. 159, 1–5 (1976). 11772 10.1042/bj1590001 PMC1164030

[b12] Wilson J. E. & Chin A. Chelation of divalent cations by ATP, studied by titration calorimetry. Anal. Biochem. 193, 16–19 (1991). 1645933 10.1016/0003-2697(91)90036-s

[b13] Kuppuraj G., Dudev M. & Lim C. Factors governing metal–ligand distances and coordination geometries of metal complexes. J. Phys. Chem. B 113, 2952–2960 (2009). 19708219 10.1021/jp807972e

[b14] Kamerlin S. C. & Warshel A. On the energetics of ATP hydrolysis in solution. J. Phys. Chem. B 113, 15692–15698 (2009). 19888735 10.1021/jp907223t

[b15] Grauffel C. & Lim C. Factors governing the bridging water protonation state in polynuclear Mg^2+^ proteins. J. Phys. Chem. B 120, 1759–1770 (2016). 26560089 10.1021/acs.jpcb.5b09323

[b16] Pecoraro V. L., Hermes J. D. & Cleland W. W. Stability constants of Mg^2+^ and Cd^2+^ complexes of adenine nucleotides and thionucleotides and rate constants for formation and dissociation of MgATP and MgADP. Biochemistry 23, 5262–5271 (1984). 6334536 10.1021/bi00317a026

[b17] Dudev T. & Lim C. A DFT/CDM study of metal-carboxylate interactions in metalloproteins: Factors governing the maximum number of metal-bound carboxylates. J. Am. Chem. Soc. 128, 1553–1561 (2006). 16448126 10.1021/ja055797e

[b18] Martell A. E. & Schwarzbach G. Adenosinphosphate und triphosphat als komplexbildner für calcium und magnesium. Helvetica Chimica Acta 39, 653–661 (1956).

[b19] O’Sullivan W. J. & Smithers G. W. Stability constants for biologically important metal-ligand complexes. Methods Enzymol. 63, 294–336 (1979). 41156 10.1016/0076-6879(79)63014-8

[b20] Jernigan R., Raghunathan G. & Bahar I. Characterization of interactions and metal ion binding sites in proteins. Curr. Opin. Struct. Biol. 4, 256–263 (1994).

[b21] Dudev M., Wang J., Dudev T. & Lim C. Factors governing the metal coordination number in metal complexes from Cambridge Structure Database analyses. J. Phys. Chem. B 110, 1889–1895 (2006). 16471760 10.1021/jp054975n

[b22] Marcus Y. Ionic radii in aqueous solutions. Chem. Rev. 88, 1475–1498 (1988).

[b23] Tunell I. & Lim C. Factors governing the metal coordination number in isolated group IA and IIA metal hydrates. Inorg. Chem. 45, 4811–4819 (2006). 16749846 10.1021/ic0519741

[b24] GaussView. Version 5 (Semichem Inc., Shawnee Mission, KS., 2009).

[b25] Allen F. H. The Cambridge structural database: a quarter of a million crystal structures and rising. Acta Crystallogr., Sect. B: Struct. Sci. B58, 380–388 (2002). 10.1107/s0108768102003890 12037359

[b26] Gaussian 09, Revision A.02 (Gaussian, Inc., Wallingford CT, 2009).

[b27] Marenich A. V., Cramer C. J. & Truhlar D. G. Universal solvation model based on solute electron density and a continuum model of the solvent defined by the bulk dielectric constant and atomic surface tensions. J. Phys. Chem. B 113, 6378–6396 (2009). 19366259 10.1021/jp810292n

[b28] McQuarrie D. A. Statistical Mechanics. (Harper and Row, 1976).

[b29] Alecu I. M., Zheng J., Zhao Y. & Truhlar D. G. Computational Thermochemistry: Scale Factor Databases and Scale Factors for Vibrational Frequencies Obtained from Electronic Model Chemistries. J. Chem. Theory Comput. 6, 2872–2887 (2010). 26616087 10.1021/ct100326h

[b30] Dudev T., Chang L.-Y. & Lim C. Factors governing the substitution of La^3+^ for Ca^2+^ and Mg^2+^ in metalloproteins: A DFT/CDM study. J. Am. Chem. Soc. 127, 4091–4103 (2005). 15771547 10.1021/ja044404t

