# Supplementary material for: How Native and Alien Metal Cations Bind ATP: Implications for Lithium as a Therapeutic Agent
Source: Sci Rep. 2017 Feb 14;7:42377. doi: 10.1038/srep42377 (PMC5307966; doi:10.1038/srep42377)
Supplement: Supplementary Information [file srep42377-s1.pdf]

How Native and Alien Metal Cations Bind ATP: Implications for Lithium as a Therapeutic Agent

Todor Dudev,[†,*] Cedric Grauffel,[‡] and Carmay Lim[‡,§,*]

[†]Faculty of Chemistry and Pharmacy, Sofia University, Sofia 1164, Bulgaria

[‡]Institute of Biomedical Sciences, Academia Sinica, Taipei 11529, Taiwan

[§]Department of Chemistry, National Tsing Hua University, Hsinchu 300, Taiwan

Table S1a. RMSD of the ATP heavy atoms between different ATP-Mg complexes

	Mg(αβγ)	Mg(αβ)	Mg(αγ)	Mg(βγ)	Mg(γγ)	Mg(α)	Mg(β)	Mg(γ)
**Mg(αβγ)**	0	1.6	0.4	1.9	2.0	1.7	1.1	1.6
**Mg(αβ)**		0	1.4	2.4	1.8	1.7	1.7	2.1
**Mg(αγ)**			0	2.0	2.1	1.6	1.4	1.8
**Mg(βγ)**				0	3.1	**3.4**	1.7	1.3
**Mg(γγ)**					0	1.4	2.3	2.8
**Mg(α)**						0	2.3	2.9
**Mg(β)**							0	1.0
**Mg(γ)**								0

Table S1b. RMSD of the ATP heavy atoms between different ATP-Li complexes

	Li(αβγ)	Li(αβ)	Li(αγ)	Li(βγ)	Li(γγ)	Li(α)	Li(β)	Li(γ)
**Li(αβγ)**	0	1.5	0.6	1.8	1.9	1.7	1.4	0.5
**Li(αβ)**		0	1.6	0.9	1.7	0.8	0.1	1.4
**Li(αγ)**			0	1.8	**2.2**	1.7	1.6	0.3
**Li(βγ)**				0	1.8	0.6	0.8	1.6
**Li(γγ)**					0	1.7	1.7	2.1
**Li(α)**						0	0.8	1.6
**Li(β)**							0	1.4
**Li(γ)**								0

Table S2a. RMSD of the ATP heavy atoms of ATP-Mg(αβγ)-Li complexes

	**Mg(αβγ)**	**Mg (αβγ) – Li(α)**	**Mg (αβγ) – Li(β)**	**Mg (αβγ) – Li(γ)**	**Mg (αβγ) – OH^{bridge}– Li(α)**	**Mg (αβγ) – OH^{bridge}– Li(β)**	**Mg (αβγ) – OH^{bridge}– Li(βγ)**	**Mg (αβγ) – OH^{bridge}– Li(γ)**
**Mg(αβγ)**	0	0.2	0.2	0.2	0.3	0.3	0.2	0.5
**Mg(αβγ) – Li(α)**		0	0.3	0.3	0.4	0.4	0.3	0.6
**Mg(αβγ) – Li(β)**			0	0.1	0.3	0.3	0.1	0.4
**Mg(αβγ) – Li(γ)**				0	0.3	0.3	0.2	0.4
**Mg(αβγ) – OH^{bridge}– Li(α)**					0	0.1	0.3	0.1
**Mg(αβγ) – OH^{bridge}– Li(β)**						0	0.6	0.2
**Mg(αβγ) – OH^{bridge}– Li(βγ)**							0	0.4
**Mg(αβγ) – OH^{bridge}– Li(γ)**								0

Table S2b. RMSD of the ATP heavy atoms of ATP-Mg(αβ)-Li / ATP-Mg(βγ)-Li complexes relative to the respective ATP-Mg(αβ)/ ATP-Mg(βγ) complex

	**Mg(αβ)**		**Mg(βγ)**
**Mg(αβ) – OH^{bridge}–Li(α)**	0.6	Mg(βγ) – OH^{bridge}–Li(α)	1.0
**Mg(αβ) – OH^{bridge}–Li(β)**	0.2	Mg(βγ) – OH^{bridge}–Li(β)	0.3
**Mg(αβ) – OH^{bridge}–Li(γ)**	0.3	Mg(βγ) – OH^{bridge}–Li(γ)	0.2
**Mg(αβ) – OH^{bridge}–Li(βγ)**	0.3	Mg(βγ) – OH^{bridge}–Li(αβ)	0.2

Table S3a. Experimental vs. computed metal–O distance in tetrahydrated Li^+.

Method			$Li–O_H$ (Å)
X-Ray:			1.94 ± 0.05
B3LYP	/	6-31+G(d,p)	1.97
B3LYP	/	6-311++G(d,p)	1.97
B3LYP	/	6-311++G(2d,2p)	1.98
SVWN	/	6-31+G(d,p)	NA
SVWN	/	6-311++G(d,p)	2.30
SVWN	/	6-311++G(2d,2p)	2.60
M062X	/	6-31+G(d,p)	1.92
**M062X**	**/**	**6-311++G(d,p)**	**1.96**
M062X	/	6-311++G(2d,2p)	1.97
M06HF	/	6-31+G(d,p)	1.91
M06HF	/	6-311++G(d,p)	1.92
M06HF	/	6-311++G(2d,2p)	1.92
BMK	/	6-31+G(d,p)	1.93
BMK	/	6-311++G(d,p)	1.94
BMK	/	6-311++G(2d,2p)	1.94

Table S3b. Experimental vs. computed geometries of the CSD entry ‘fecwit’:

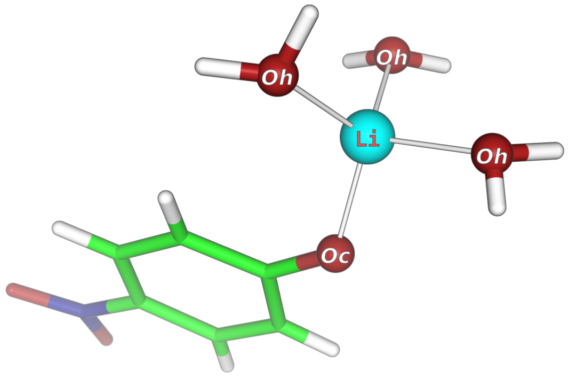

Method			Li–O_H (Å)	Li–O_C (Å)
X-Ray:			1.97	1.88
**M062X**	**/**	**6-311++G(d,p)**	**1.95**	**1.85**
M062X	/	6-311++G(2d,2p)	1.95	1.83
M062X	/	6-31+G(d,p)	1.96	1.85
M062X	/	6-31+G(2d,2p)	1.95	1.83
B3LYP	/	6-311++G(d,p)	2.00	1.86
B3LYP	/	6-311++G(2d,2p)	2.01	1.86
B3LYP	/	6-31+G(d,p)	2.00	1.86
B3LYP	/	6-31+G(2d,2p)	2.00	1.86
SVWN	/	6-311++G(d,p)	2.09	1.83
SVWN	/	6-311++G(2d,2p)	2.44	1.86
SVWN	/	6-31+G(d,p)	3.09	1.89
SVWN	/	6-31+G(2d,2p)	3.11	1.88
BMK	/	6-311++G(d,p)	1.96	1.83
BMK	/	6-311++G(2d,2p)	1.96	1.86
BMK	/	6-31+G(d,p)	1.96	1.83
BMK	/	6-31+G(2d,2p)	1.96	1.82
M06HF	/	6-311++G(d,p)	NA	NA
M06HF	/	6-311++G(2d,2p)	1.95	1.87
M06HF	/	6-31+G(d,p)	1.95	1.83
M06HF	/	6-31+G(2d,2p)	1.95	1.83

Table S3c. Experimental vs. computed geometries of the CSD entry 'ejezup':

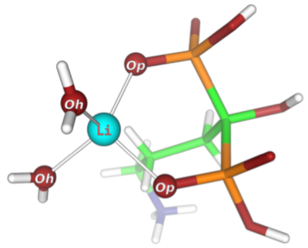

Method			Li-O_p (Å)	Li-O_w (Å)
X-Ray:			1.90	1.92
B3LYP	/	6-311++G(d,p)	1.89	2.01
B3LYP	/	6-31+G(d)	1.88	2.02
B3LYP	/	6-31+G(d,p)	1.89	2.02
B3LYP	/	6-31+G(2d,2p)	1.88	2.02
B3LYP	/	6-31+G(3d,p)	1.88	2.02
B3LYP	/	6-311++G(2d,2p)		
SVWN	/	6-311++G(d,p)	1.91	2.60
SVWN	/	6-31+G(d)	1.90	2.60
SVWN	/	6-31+G(d,p)	1.90	2.56
SVWN	/	6-31+G(2d,2p)	1.90	2.59
SVWN	/	6-31+G(3d,p)	1.86	2.58
SVWN	/	6-311++G(2d,2p)	1.91	2.60
**M062X**	**/**	**6-311++G(d,p)**	**1.87**	**1.97**
M062X	/	6-31+G(d)	1.87	2.07
M062X	/	6-31+G(d,p)	1.87	2.02
M062X	/	6-31+G(2d,2p)	1.93	2.04
M062X	/	6-31+G(3d,p)	1.84	1.96
M062X	/	6-311++G(2d,2p)	1.90	2.01
M06HF	/	6-311++G(d,p)	1.93	1.97
M06HF	/	6-31+G(d)	1.93	1.96
M06HF	/	6-31+G(d,p)	1.93	1.96
M06HF	/	6-31+G(2d,2p)	1.92	1.97
M06HF	/	6-31+G(3d,p)	1.90	1.98
M06HF	/	6-311++G(2d,2p)	1.92	1.97
BMK	/	6-311++G(d,p)	1.89	1.98
BMK	/	6-31+G(d)	1.86	1.98
BMK	/	6-31+G(d,p)	1.86	1.97
BMK	/	6-31+G(2d,2p)	1.85	1.98
BMK	/	6-31+G(3d,p)	1.87	1.97
BMK	/	6-311++G(2d,2p)	1.89	1.97

Table S3d. Experimental vs. computed metal–O distance in hexahydrated Mg^{2+}:

Method			Mg–O (Å)
X-Ray:			2.07 ± 0.03
B3LYP	/	6-31+G(d)	2.09
B3LYP	/	6-31+G(d,p)	2.09
B3LYP	/	6-31+G(2d,2p)	2.08
B3LYP	/	6-31+G(3d,p)	2.08
B3LYP	/	6-31+G(3d,2p)	2.07
B3LYP	/	6-311++G(d,p)	2.08
B3LYP	/	6-311++G(2d,2p)	2.08
SVWN	/	6-31+G(d)	2.04
SVWN	/	6-31+G(d,p)	2.03
SVWN	/	6-31+G(2d,2p)	2.02
SVWN	/	6-31+G(3d,p)	2.02
SVWN	/	6-31+G(3d,2p)	2.02
SVWN	/	6-311++G(d,p)	2.03
SVWN	/	6-311++G(2d,2p)	2.02
M062X	/	6-31+G(d)	2.05
M062X	/	6-31+G(d,p)	2.04
M062X	/	6-31+G(2d,2p)	2.03
M062X	/	6-31+G(3d,p)	2.04
M062X	/	6-31+G(3d,2p)	2.03
**M062X**	**/**	**6-311++G(d,p)**	**2.04**
M062X	/	6-311++G(2d,2p)	2.03
M06HF	/	6-31+G(d)	2.05
M06HF	/	6-31+G(d,p)	2.05
M06HF	/	6-31+G(2d,2p)	2.04
M06HF	/	6-31+G(3d,p)	2.04
M06HF	/	6-31+G(3d,2p)	2.04
M06HF	/	6-311++G(d,p)	2.05
M06HF	/	6-311++G(2d,2p)	2.04
BMK	/	6-31+G(d)	2.06
BMK	/	6-31+G(d,p)	2.06
BMK	/	6-31+G(2d,2p)	2.05
BMK	/	6-31+G(3d,p)	2.04
BMK	/	6-31+G(3d,2p)	NA
BMK	/	6-311++G(d,p)	2.05
BMK	/	6-311++G(2d,2p)	2.05

Table S3e. Experimental vs. computed geometries of the CSD entry 'geqbio':

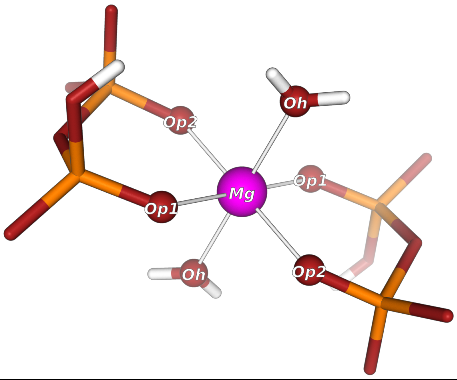

Method			Mg–O$_H$ (Å)	Mg–O$_{P1}$ (Å)	Mg–O$_{P2}$ (Å)	Mg–O$_{P1}$–P (°)	Mg–O$_{P2}$–P (°)
X-Ray:			2.13	2.09	2.05	133.5	133.5
B3LYP	/	6-31+G(d)	2.21	2.08	2.08	125.8	133.8
B3LYP	/	6-31+G(d,p)	2.22	2.08	2.08	125.8	133.4
B3LYP	/	6-31+G(2d,2p)	2.22	2.07	2.07	126.0	133.3
B3LYP	/	6-31+G(3d,p)	2.22	2.07	2.07	126.4	133.3
B3LYP	/	6-31+G(3d,2p)	2.23	2.06	2.07	126.4	133.2
B3LYP	/	6-311++G(d,p)	2.22	2.08	2.08	127.3	134.2
B3LYP	/	6-311++G(2d,2p)	2.22	2.07	2.07	126.2	133.2
SVWN	/	6-31+G(d)	2.11	2.06	2.04	135.1	122.0
SVWN	/	6-31+G(d,p)	2.11	2.06	2.04	134.9	121.8
SVWN	/	6-31+G(2d,2p)	2.12	2.05	2.03	132.2	124.0
SVWN	/	6-31+G(3d,p)	2.11	2.05	2.02	133.6	123.3
SVWN	/	6-31+G(3d,2p)	2.12	2.05	2.02	132.7	123.8
SVWN	/	6-311++G(d,p)	2.12	2.06	2.04	135.5	122.9
SVWN	/	6-311++G(2d,2p)	2.12	2.04	2.06	122.2	130.8
M062X	/	6-31+G(d)	2.14	2.07	2.04	135.6	123.4
M062X	/	6-31+G(d,p)	2.14	2.08	2.04	135.6	123.0
M062X	/	6-31+G(2d,2p)	2.14	2.06	2.03	134.8	123.6
M062X	/	6-31+G(3d,p)	2.14	2.06	2.03	134.7	123.7
M062X	/	6-31+G(3d,2p)	2.15	2.05	2.03	134.7	123.8
**M062X**	**/**	**6-311++G(d,p)**	**2.15**	**2.07**	**2.04**	**136.5**	**124.4**
M062X	/	6-311++G(2d,2p)	2.14	2.06	2.03	135.2	123.6
M06HF	/	6-31+G(d)	2.15	2.07	2.07	124.3	132.8
M06HF	/	6-31+G(d,p)	2.15	2.07	2.07	124.1	132.7
M06HF	/	6-31+G(2d,2p)	2.15	2.06	2.05	124.2	131.8
M06HF	/	6-31+G(3d,p)	2.15	2.06	2.05	124.5	132.1
M06HF	/	6-31+G(3d,2p)	2.15	2.06	2.05	124.3	132.0
M06HF	/	6-311++G(d,p)	2.15	2.06	2.06	125.6	133.5
M06HF	/	6-311++G(2d,2p)	2.15	2.06	2.05	124.8	132.3
BMK	/	6-31+G(d)	2.16	2.09	2.05	135.9	124.3
BMK	/	6-31+G(d,p)	2.18	2.06	2.06	128.1	129.7

BMK	/	6-31+G(2d,2p)	NA	NA	NA	NA	NA
BMK	/	6-31+G(3d,p)	2.18	2.04	2.04	129	130.5
BMK	/	6-31+G(3d,2p)	NA	NA	NA	NA	NA
BMK	/	6-311++G(d,p)	2.17	2.08	2.04	136.7	125.6
BMK	/	6-311++G(2d,2p)	2.18	2.05	2.05	128.5	130.1

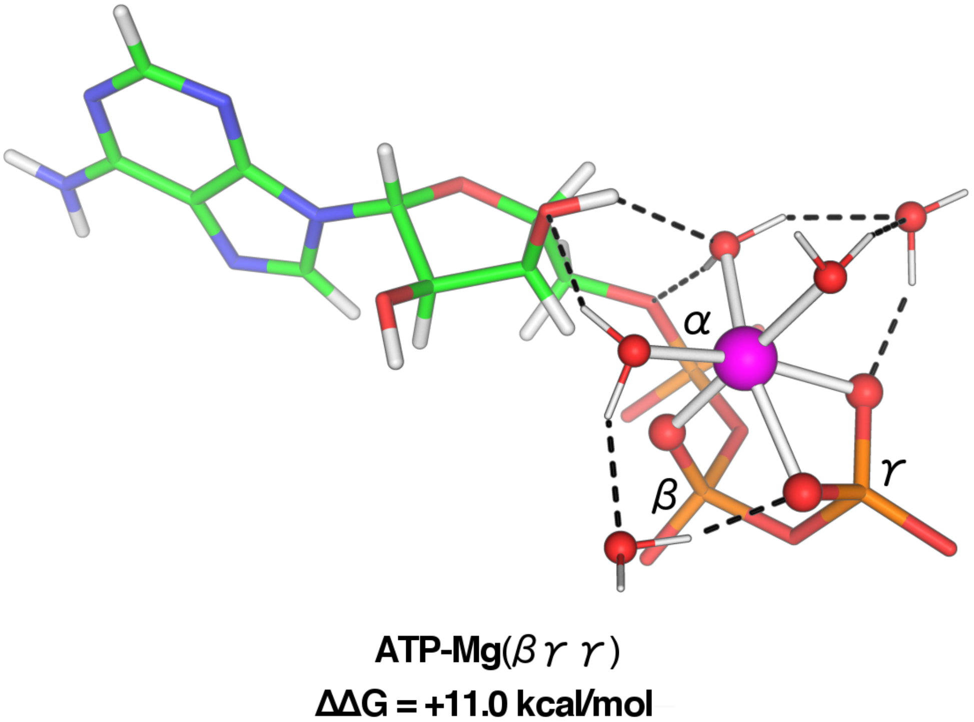

Figure S1. M062X/6-311++G(d,p)-optimized structure of ATP–Mg(βγγ).

A

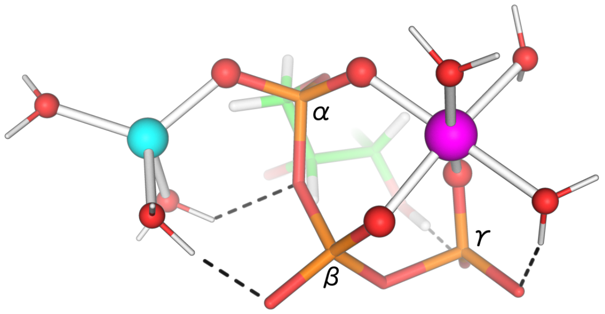

ATP-Mg(αβγ)-Li(α)
ΔΔG = +26.1 kcal/mol

B

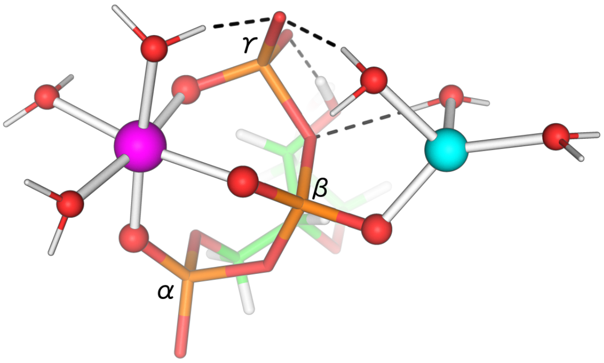

ATP-Mg(αβγ)-Li(β)
ΔΔG = +25.6 kcal/mol

C

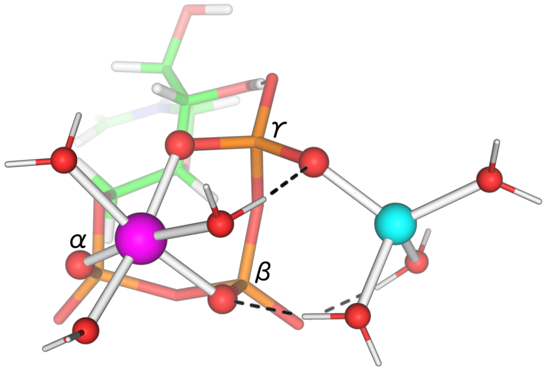

ATP-Mg(αβγ)-Li(γ)
ΔΔG = +23.5 kcal/mol

Figure S2. M062X/6-311++G(d,p)-optimized structures of Li^+ monodentately bound to ATP-Mg-αβγ

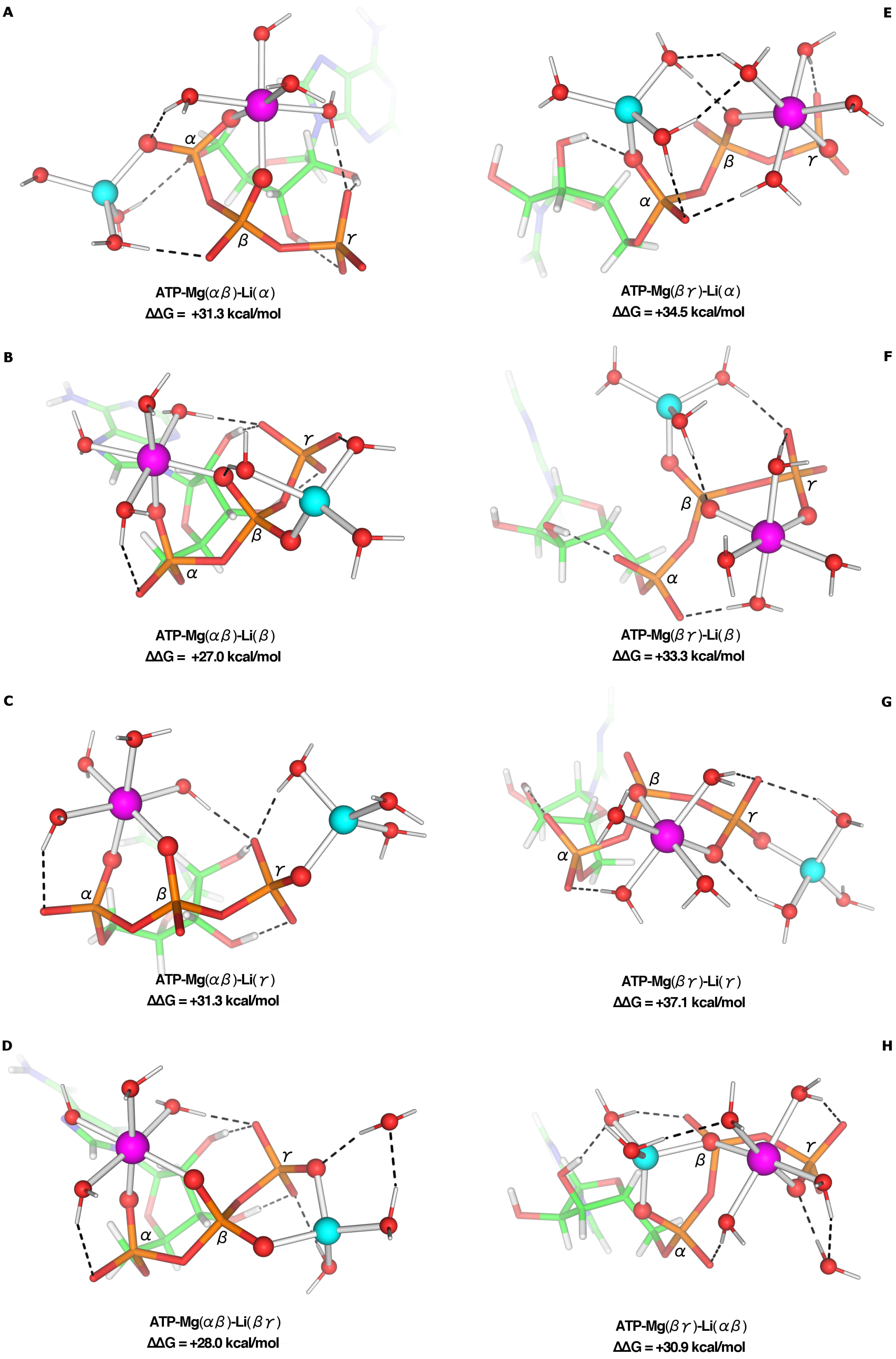

Figure S3. M062X/6-311++G(d,p)-optimized structures of ATP-Mg-αβ-Li or ATP-Mg-βγ-Li mononuclear complexes

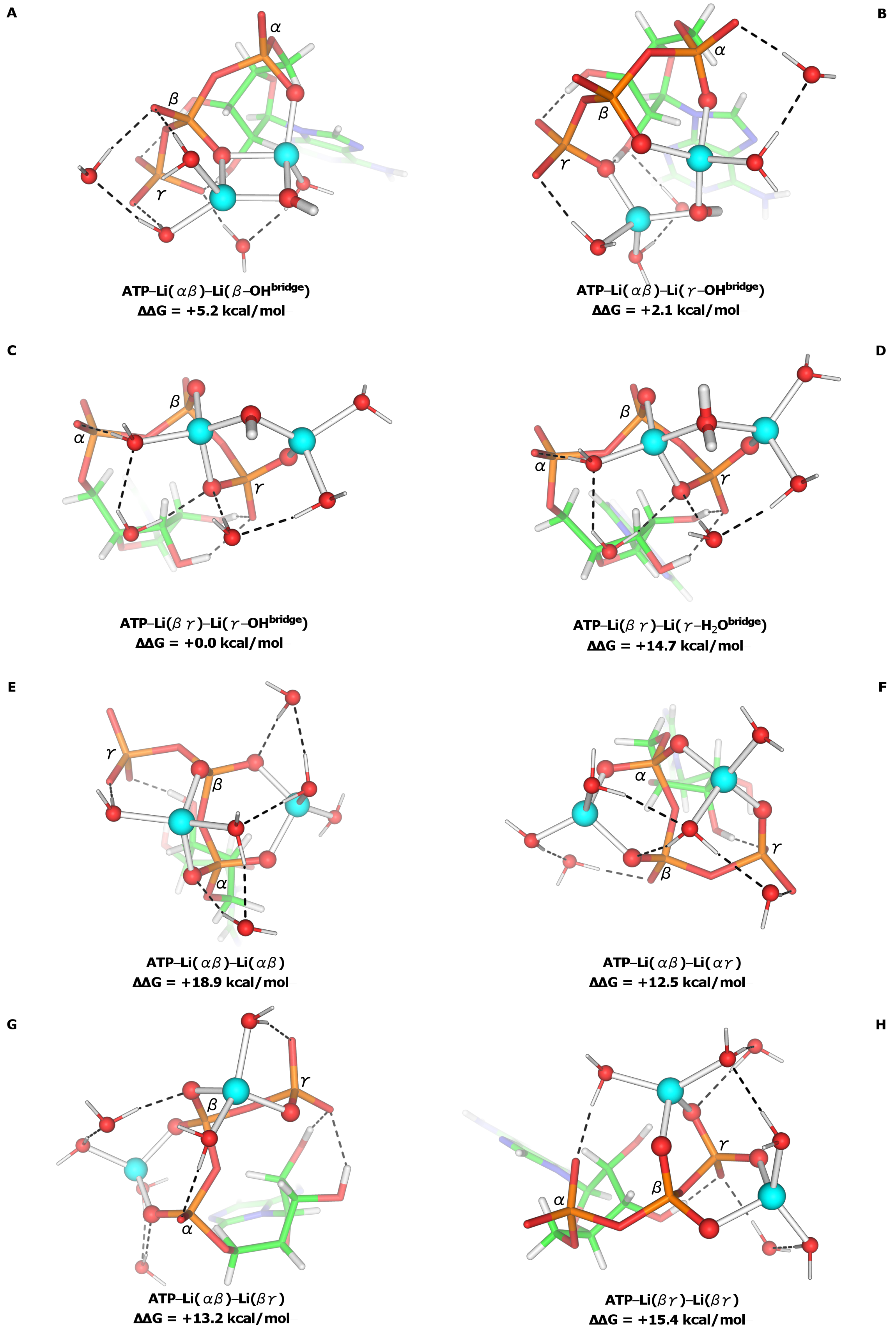

Figure S4. M062X/6-311++G(d,p)-optimized structures of $[Li_2(H_2O)_6ATP]^{2-}$.
